# Body fluids should be identified before estimating the time since deposition (TsD) in microbiome-based stain analyses for forensics

**DOI:** 10.1128/spectrum.02480-23

**Published:** 2024-03-12

**Authors:** Jun Zhang, Daijing Yu, Tian Wang, Niu Gao, Linyu Shi, Yaya Wang, Yumei Huo, Zhimin Ji, Junli Li, Xiaomeng Zhang, Liwei Zhang, Jiangwei Yan

**Affiliations:** 1 School of Forensic Medicine, Shanxi Medical University, Jinzhong, Shanxi, China; 2 Shanxi Key Laboratory of Forensic Medicine, Jinzhong, Shanxi, China; American Type Culture Collection, Manassas, Virginia, USA

**Keywords:** microbial characteristics, body fluid identification, time since deposition estimation, mutual influence, codetection

## Abstract

Identification and the time since deposition (TsD) estimation of body fluid stains from a crime scene could provide valuable information for solving the cases and are always difficult for forensics. Microbial characteristics were considered as a promising biomarker to address the issues. However, changes in the microbiota may damage the specific characteristics of body fluids. Correspondingly, incorrect body fluid identification may result in inaccurate TsD estimation. The mutual influence is not well understood and limited the codetection. In the current study, saliva, semen, vaginal secretion, and menstrual blood samples were exposed to indoor conditions and collected at eight time points (from fresh to 30 days). High-throughput sequencing based on the 16S rRNA gene was performed to characterize the microbial communities. The results showed that a longer TsD could decrease the discrimination of different body fluid stains. However, the accuracies of identification still reached a quite high value even without knowing the TsD. Correspondingly, the mean absolute error (MAE) of TsD estimation significantly increased without distinguishing the types of body fluids. The predictive TsD of menstrual blood reached a quite low MAE (1.54 ± 0.39 d). In comparison, those of saliva (6.57 ± 1.17 d), semen (6.48 ± 1.33 d), and vaginal secretion (5.35 ± 1.11 d) needed to be further improved. The great effect of individual differences on these stains limited the TsD estimation accuracy. Overall, microbial characteristics allow for codetection of body fluid identification and TsD estimation, and body fluids should be identified before estimating TsD in microbiome-based stain analyses.

**IMPORTANCE:**

Emerged evidences suggest microbial characteristics could be considered a promising tool for identification and time since deposition (TsD) estimation of body fluid stains. However, the two issues should be studied together due to a potential mutual influence. The current study provides the first evidence to understand the mutual influence and determines an optimal process for codetection of identification and TsD estimation for unknown stains for forensics. In addition, we involved aged stains into our study for identification of body fluid stains, rather than only using fresh stains like previous studies. This increased the predictive accuracy. We have preliminary verified that individual differences in microbiotas limited the predictive accuracy of TsD estimation for saliva, semen, and vaginal secretion. Microbial characteristics could provide an accurate TsD estimation for menstrual blood. Our study benefits the comprehensive understanding of microbiome-based stain analyses as an essential addition to previous studies.

## INTRODUCTION

Body fluid identification and determination of the time since deposition (TsD) are crucial complements for forensics to assess the relevance of stains from crime scenes and are helpful for crime scene investigation and reconstruction (1, 2). Body fluid identification is commonly performed in forensic labs with conventional approaches by testing a chemical reaction of specific enzyme catalysis (3). The tests are fast and visually detected but have a high error rate. Furthermore, the methods can not provide any information about TsD of body fluids. Spectroscopy, chromatography, and electron spin resonance have been explored for TsD determination of blood stains (4) but are useless for determining TsD of white or nearly colorless body fluids such as saliva, semen, and vaginal secretion. In recent years, several novel approaches by measuring gene expression of messenger RNA (mRNA) (5–8), microRNA (miRNA) (9, 10), and epigenetic markers (11, 12) have been reported to be used for body identification. The degradation patterns of mRNA examined through quantitative PCR could be used to predict the TsD of body fluids (13, 14).

In addition, microbiome-based stain analyses have emerged as a new research area to demonstrate microbial characteristics to identify body fluids and estimate the TsD of stains (15). The number of microbial cells is greater than that of human cells in the human body (16). These microbiotas contain abundant genomic information and even exceed the human genome (17). The results suggested that microbiotas from body fluids can still be detectable when human DNA or RNA are in low biomass or degraded.

Metagenomic information varies across body habitats (18). Therefore, the human microbiome is considered a potential tool to recognize the original body sites of biological materials. Hanssen et al. (19) collected large bacterial 16S sequencing data sets from the American Gut Project and the Human Microbiome Project (HMP) to verify the feasibility of using microbial composition data for body fluid identification. The results suggested that the optimal prediction accuracy was close to 98%. López et al. (20) trained deep learning networks containing sequencing data of 1,636 skin, oral, and vaginal samples from the HMP to classify these tissues. The values of the area under the curve were above 0.99. Most of the aged mock casework samples could still be correctly identified in the study of López et al. (20), although the prediction accuracy decreased in the aged mock casework samples compared with fresh samples (20). In another study, skin, saliva, vaginal fluid, menstrual blood, and semen samples were exposed to indoor conditions for 30 days, and the results showed that these samples were grouped by body site mainly based on the microbial community (21). Nevertheless, a few outliers that could not be correctly identified were observed in the study. These two studies suggested that specific microbial characteristics of tissue or fluid can be maintained for a period of time after tissue or fluid leaves the body. This provides us with a microbiome tool for recognizing body fluid stains left at crime scenes. The TsD of stains seems to have some effects on the microbial characteristics of specific tissues or fluids, causing a reduction in the accuracy of identification. However, previous studies were still insufficient to assess the exact effect of the TsD on body fluid identification based on microbial characteristics.

On the other hand, the microbiome was also considered a promising tool for predicting the TsD of body fluid stains. Environmental changes and the breeding of environmental microorganisms may alter the microbial community structure of the body fluid that leaves the original body site. Wang et al. (1) analyzed 16S rRNA gene sequencing data of saliva stains exposed to indoor conditions for up to 20 days, and the results suggested that the mean absolute deviation of TsD prediction was 1.41 days. Salzmann et al. (22) examined RNA sequencing data from blood, menstrual blood, saliva, semen, and vaginal secretion ranging from fresh to 1.5 years. They found that environmental bacteria altered the microbial composition of body fluid stains. The changing microbial community could be evaluated for TsD prediction. These studies predicted the TsD of stains under the condition that the type of body fluid was known. In fact, the type of body fluid at the crime scene is often unknown. Microbial successions of various body fluids might be very different. The effect of body fluid identification on TsD prediction is still unclear.

In summary, body fluid identification and TsD estimation of stains based on microbial characteristics were investigated separately in previous studies. The feasibility of simultaneous body fluid identification and TsD estimation has not been well explored. In addition, changes in the microbial community of stains provide a chance to predict TsD but may damage the specific characteristics used for body fluid identification simultaneously. Incorrect body fluid identification may also result in inaccurate TsD estimation. Their mutual influence needs further exploration. This problem determines which process should take precedence in microbiome-based stain analyses for forensics.

To investigate the problem, saliva, semen, vaginal secretion, and menstrual blood samples were exposed to indoor conditions ranging from fresh to 1 month. Microbial communities of body fluid stains were characterized by targeted 16S rRNA gene high-throughput sequencing at eight different time points. The mutual influences between body fluid identification and TsD prediction were measured to provide a reliability assessment and determine an optimal process for microbiome-based analyses for unknown stains at crime scenes.

## RESULTS

### Microbial community compositions of various body fluids at different TsD values

In the current study, a total of 192 samples of body fluids were sequenced and produced 14,990,142 high-quality (above Q20) sequences. We identified 12,347 amplicon sequence variants (ASVs) from these sequences. The sequence of each sample was rarified to an even depth of 44,676 (using “single_rarefaction.py” script in QIIME1 based on the minimum value of all samples) to normalize the ASV table.

**Fig 1:**
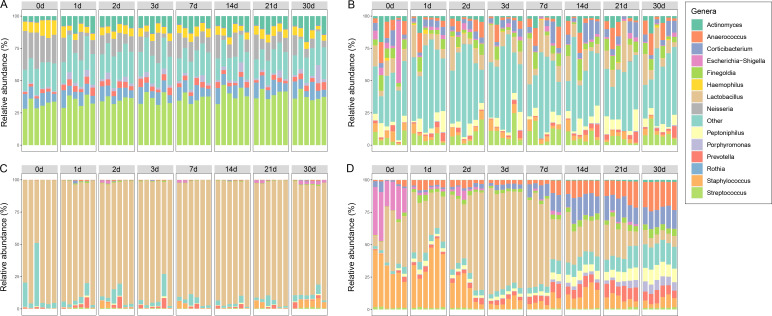
Relative abundance of bacterial genera in saliva (**A**), semen (**B**), vaginal secretion (**C**), and menstrual blood (**D**) across 30 days of exposure.

We investigated the microbial compositions of four body fluids at eight time points at the phylum (Fig. S1) and genus (Fig. 1) levels. Firmicutes was the dominant taxon in the four body fluids at the phylum level. The highest relative abundance of Firmicutes was observed in vaginal secretion. Actinobacteria, Bacteroidetes, and Proteobacteria were important taxa in saliva, semen, and menstrual blood. In these three body fluids, a higher relative abundance of Proteobacteria and lower relative abundances of Actinobacteria and Bacteroidetes were observed in fresh body fluids than in aged stains (from Day 1 to Day 30).

*Streptococcus* was the dominant genus in saliva (Fig. 1A). Fresh saliva contained the lowest relative abundances of *Streptococcus*, *Actinomyces*, and *Prevotella* and the highest relative abundances of *Haemophilus* and *Neisseria*. Semen contained the most genera and lacked an obvious dominant genus in the microbial community composition (Fig. 1B). *Lactobacillus* was the obvious dominant genus in vaginal secretion, and its mean relative abundance reached 89.74%. A significant increase was observed in *Escherichia-Shigella* (Wilcoxon test, *P* = 0.041) and *Staphylococcus* (Wilcoxon test, *P* = 0.004) in vaginal secretion at Day 30 compared with the fresh fluids (Fig. 1C). The clearest trend of microbial succession was observed in menstrual blood (Fig. 1D). *Escherichia-Shigella* and *Staphylococcus* decreased (Kruskal–Wallis test, *P* < 0.001), while *Anaerococcus*, *Corticibacterium*, *Finegoldia*, *Peptoniphilus*, *Porphyromonas*, and *Prevotella* increased (Kruskal–Wallis test, *P* < 0.001) in relative abundance from the fresh stage to aged stages. *Lactobacillus* first increased and then decreased, and its highest relative abundance was observed at Day 3.

### The effects of different factors on the microbial community compositions of body fluids

**Fig 2:**
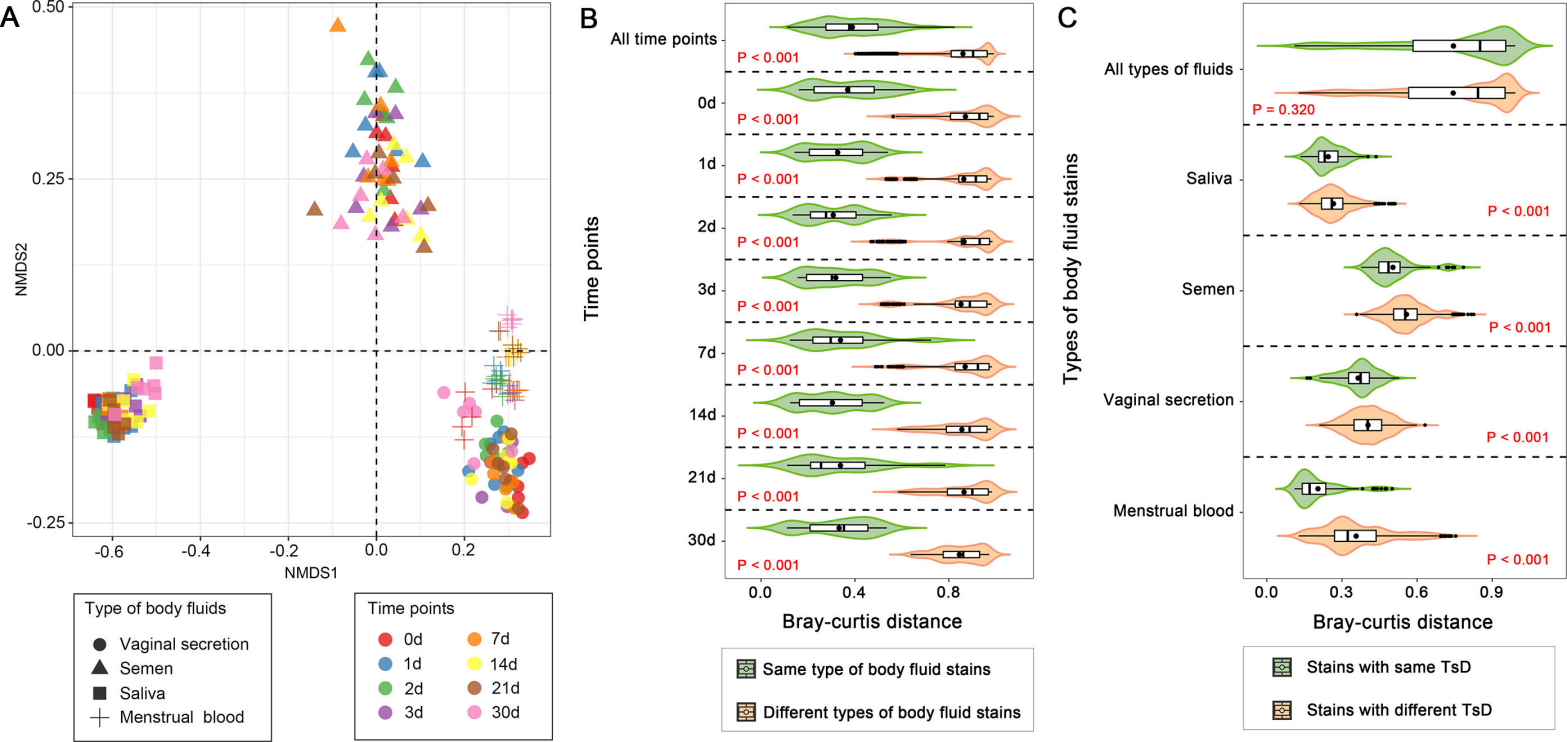
The effects of body fluid origins and TsD on microbial community structures of body fluid stains. NMDS of Bray–Curtis distances between samples is shown in A. Bray‒Curtis distances of samples from the same type and from different types of body fluids for all time points (without distinguishing TsD) and each time point are shown in B. Bray–Curtis distances of samples with the same TsD and with different TsD for all types of body fluids (without distinguishing types of body fluids) and each type of body fluid are shown in C. Statistical differences were analyzed using the Wilcoxon tests.

The result of nonmetric multidimensional scaling (NMDS) showed that samples clustered mainly based on their original body sites rather than the TsD of body fluids. Semen samples were more heterogeneous than other types of body fluids. Menstrual blood and vaginal secretion were very similar compared with the two other body fluids (Fig. 2A). The result of PERMANOVA showed that original body sites explained most of the variation (*R*^2^ = 0.694, *P* < 0.001) in the microbial community of body fluids. TsD had a small but significant effect (*R*^2^ = 0.012, *P* < 0.001) on the microbial community structure of body fluids. In addition, the interaction of original body sites and TsD explained 0.039 (*P* < 0.001) of the variation (Table 1).

**TABLE 1:** PERMANOVA tests of the effects of type of body fluid, TsD, and their interaction on microbial beta diversity between samples

Factors	*R* ^2^	*P*
**Type**	0.689	<0.001
**TSD**	0.024	<0.001
**Type: TsD**	0.068	<0.001
**Residual**	0.217	

The Bray–Curtis distances between samples of the same body fluid were significantly (*P* < 0.001) lower than those of different body fluids for each sampling time point. A similar result was observed in the merged data that included all samples from different time points (Fig. 2B). This suggested that we could still identify the body fluids effectively without distinguishing time points. Significant differences (*P* < 0.001) in Bray–Curtis distance were observed between samples from the same day and different days for each type of body fluid. However, the Bray–Curtis distances of samples from the same day and different days almost overlapped without distinguishing the types of body fluids (Fig. 2C). This suggested that we could not distinguish whether the samples were from the same day without knowing the type of body fluids.

**Fig 3:**
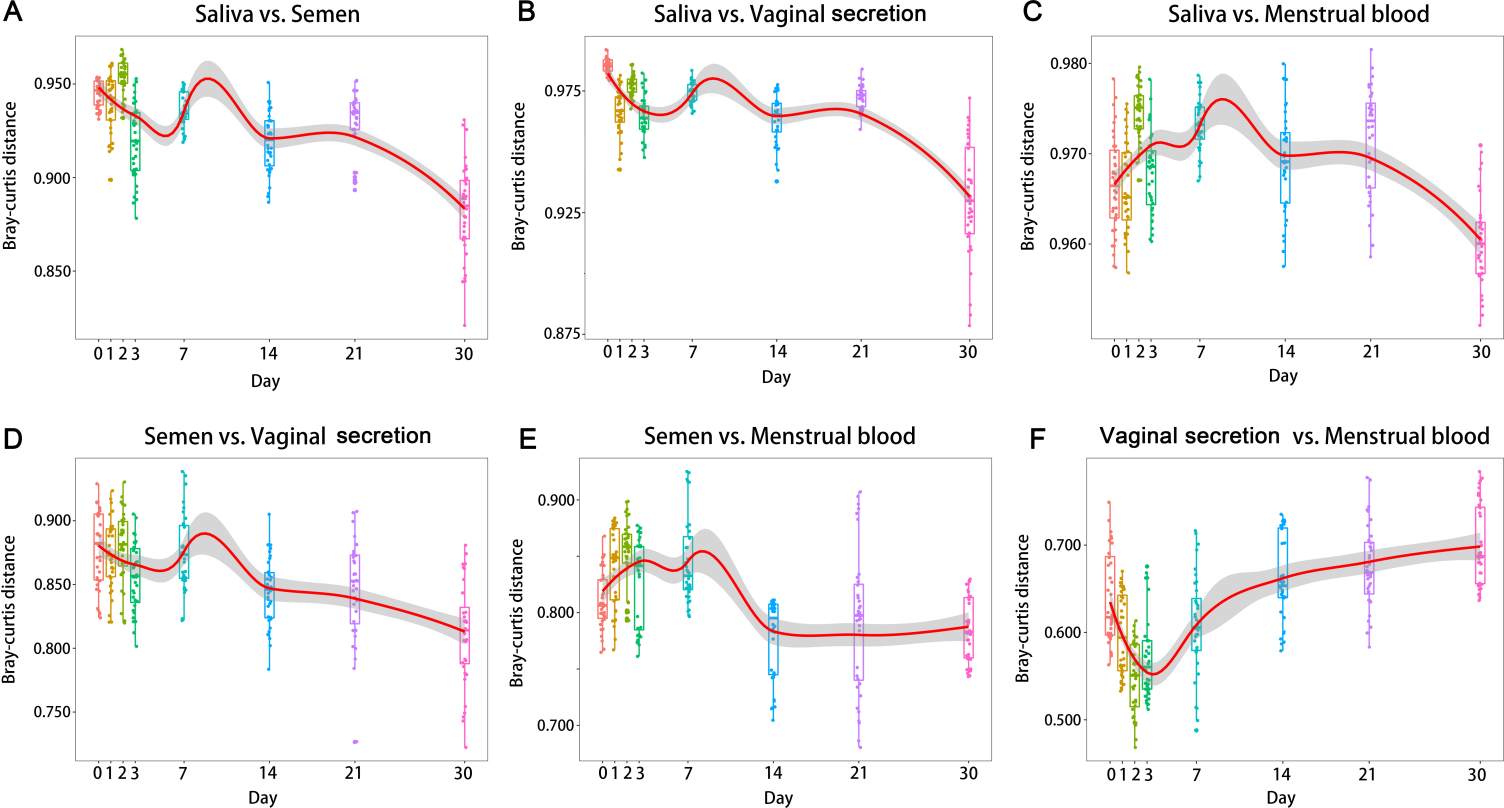
Pairwise comparisons (Bray–Curtis distance) between different types of body fluids across 30 days of exposure.

We also investigated the Bray–Curtis distance between pairwise types of body fluids varying in TsD (Fig. 3). The results showed that the distance of the microbial community between vaginal secretion and menstrual blood decreased from fresh to Day 2 and then increased from Day 2 to Day 30. The greatest value was observed at Day 30 (Fig. 3F). The changing pattern was very different from other combinations of pairwise comparisons. The smallest value was observed at Day 30 for those comparisons (Fig. 3A through E). This suggested that exposure for 30 days decreased (Wilcoxon tests, *P* < 0.001) the discrimination between different body fluids except for between vaginal secretion and menstrual blood. In addition, the Bray–Cutis distances between vaginal secretion and menstrual blood were smaller (Wilcoxon tests, *P* < 0.001) than those of other comparisons.

The effect of TsD on the microbial community structure for each body fluid was investigated in our study. In addition, the effect of individual differences (ID) was also measured. The results showed that individual differences explained a greater variation in microbial variation than TsD in saliva, semen, and vaginal secretion. In contrast, TsD explained more variation than individual differences in the microbial community of menstrual blood (Table 2; Fig. S2).

**TABLE 2:** PERMANOVA test of the effects of type of TsD, ID, and their interaction on microbial beta diversity between samples for saliva, semen, vaginal secretion, and menstrual blood, respectively

Factors	Saliva		Semen		Vaginal secretion		Menstrual blood	
	*R* ^2^	*P*	*R* ^2^	P	*R* ^2^	*P*	*R* ^2^	*P*
**TSD**	0.077	<0.001	0.036	0.026	0.046	0.034	0.427	<0.001
**ID**	0.471	<0.001	0.212	<0.001	0.519	<0.001	0.021	1.000
**TSD:ID**	0.058	0.380	0.095	0.400	0.042	0.642	0.013	1.000
**Residual**	0.395		0.657		0.393		0.539	

Significant (*P* < 0.001) linear relationships between similarities of microbial communities and intervals of TsD were observed in all four body fluids (Fig. S3). This suggested that the microbial communities of these body fluids changed with a regular pattern and provided a potential chance to predict TsD. The steepest slope was observed in menstrual blood, suggesting the fastest rate of microbial succession.

### Body fluid identification and TsD prediction based on microbial community characteristics

**Fig 4:**
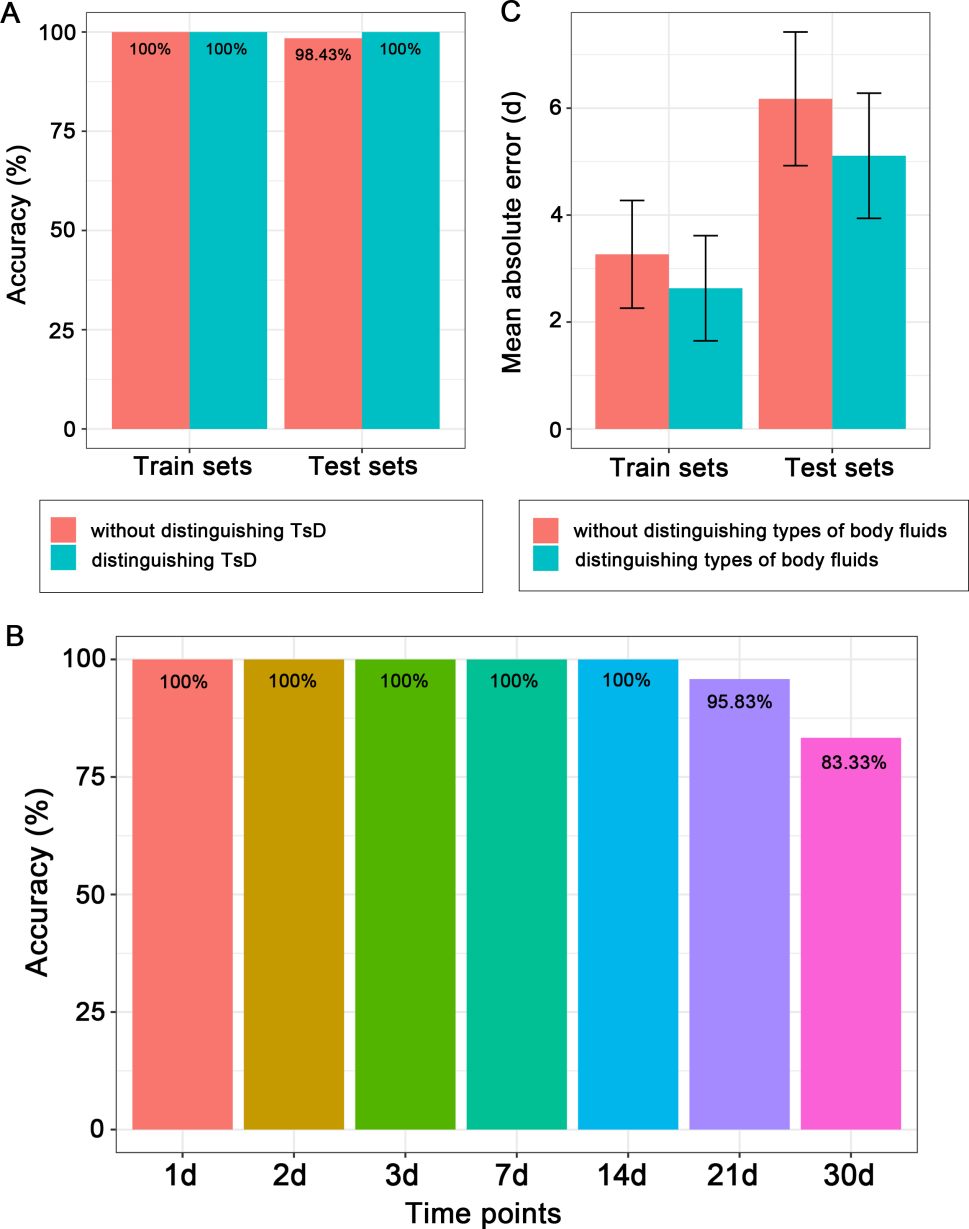
The accuracies of predictive models for body fluid identification (**A and B**) and TsD estimation (**C**). The predictive accuracies for body fluid identification performed with samples of all time points (without distinguishing TsD) and each time point are shown in A. The accuracies of predictive models constructed with fresh samples to recognize aged body fluids are shown in B. The predictive accuracies for TsD estimation performed with samples of all types of body fluids (without distinguishing types of body fluids) and each type of body fluid are shown in C.

The random forest algorithm was used to construct initial predictive models and select microbial biomarkers. Training sets were used to construct the models, and both training and testing sets were used to test the accuracies of the models. The results showed that the accuracies of body fluid identification reached an extreme value (training sets: 100.00%; testing sets: 98.43%) even without knowing the TsD (Fig. 4A). In addition, fresh body fluids (Day 0) were used to train the model to recognize the types of aged body fluid stains (from Day 1 to Day 30). The results showed that the predictive accuracies for samples collected from Day 1 to Day 14 reached 100.00%. Then, the accuracies decreased at Day 21 (95.83%) and Day 30 (83.33%). This suggested that the model that was only constructed with fresh body fluid data was insufficient to reach a high identification accuracy for aged body fluid stains (Fig. 4B). The accuracies of TsD prediction significantly decreased without distinguishing the types of body fluids, regardless of whether training sets (Wilcoxon test, *P* = 0.004) or testing sets (Wilcoxon test, *P* = 0.032) were used (Fig. 4C).

**Fig 5:**
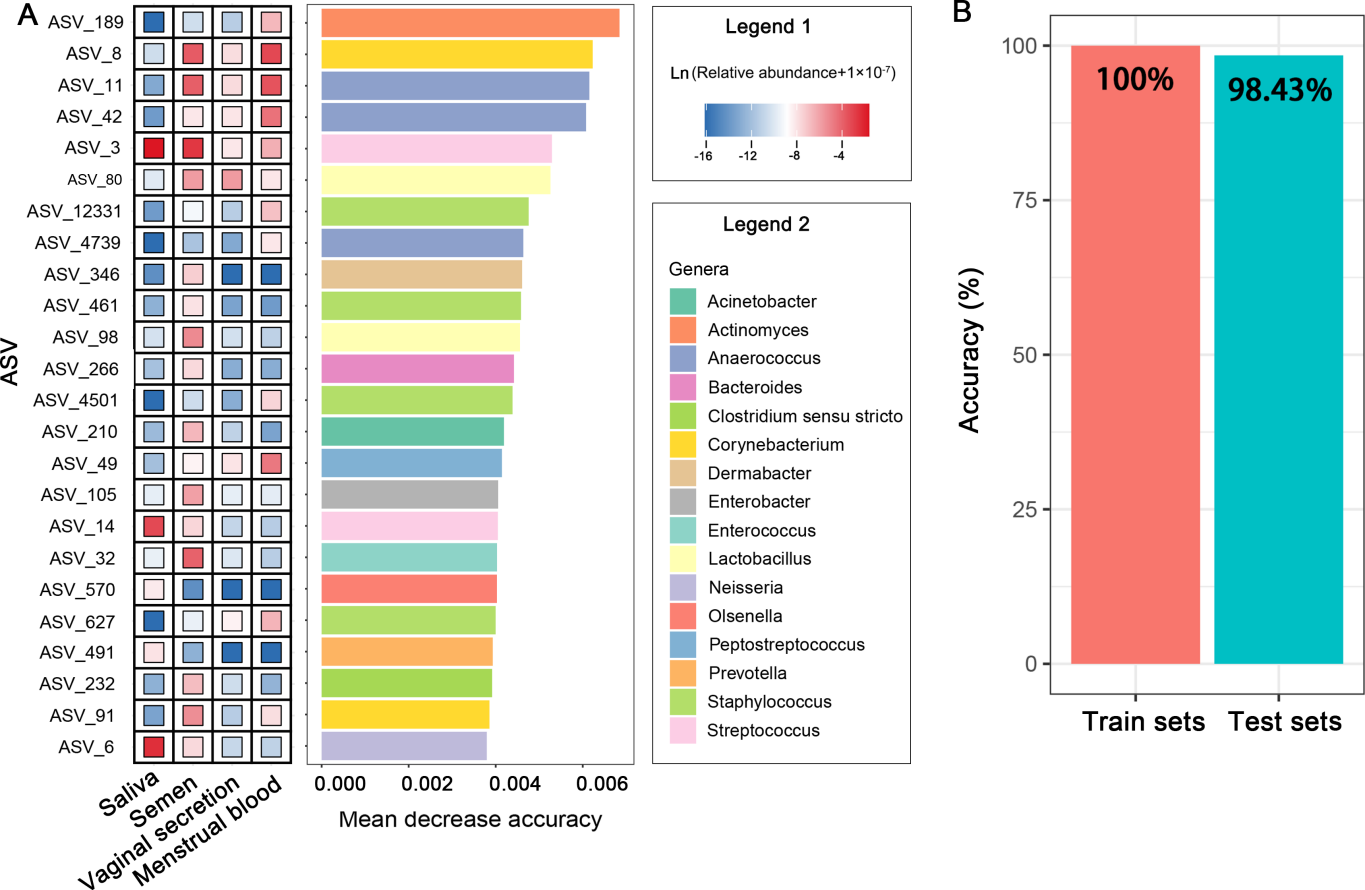
The optimal biomarkers were selected to construct final predictive models for body fluid identification and TsD estimation. The optimal biomarkers selected for body fluid identification are shown in A. The predictive accuracies for body fluid identification performed with optimal biomarkers are shown in B.

Therefore, the data used to construct the model of body fluid identification should be collected from various TsD values. However, the TsD prediction model should be constructed separately for each body fluid. Microbial biomarkers were selected based on the minimum error of 10-fold cross-validation with five repeats to construct final prediction models. Finally, 24 ASVs were selected as the optimal biomarker set to construct the model of body fluid identification (Fig. 5A; Fig. S4). Among these biomarkers, ASV_189 belonging to *Actinomyces* was the most important taxon for classification, followed by ASV_8 belonging to *Corynebacterium* and ASV_11 and ASV_42 belonging to *Anaerococcus*. The highest and lowest relative abundances of these taxa were observed in menstrual blood and saliva, respectively. ASV_3 and ASV_14, belonging to *Streptococcus*, had the highest relative abundance in saliva (Fig. 5A).

In addition, 9 ASVs, 677 ASVs, 296 ASVs, and 48 ASVs were selected as the optimal biomarker sets to construct the models of TsD prediction for saliva, semen, vaginal secretion, and menstrual blood, respectively. The most important taxa of the top 20 are ranked. ASV_61 belonging to *Fusobacterium*, ASV_376 belonging to *Clostridium sensu stricto 1*, and ASV_1172 belonging to *Dialister* were the most important taxa to predict TsD of saliva (Fig. S5A), semen (Fig. S5B), and vaginal secretion (Fig. S5C), respectively. These taxa decreased from fresh to Day 30 in the corresponding body fluids. ASV_78, belonging to *Porphyromonas*, was the most important taxon to predict TsD of menstrual blood. Interestingly, all of the most important taxa ranked in the top 20 increased from fresh to Day 30 in menstrual blood (Fig. S5D).

**Fig 6:**
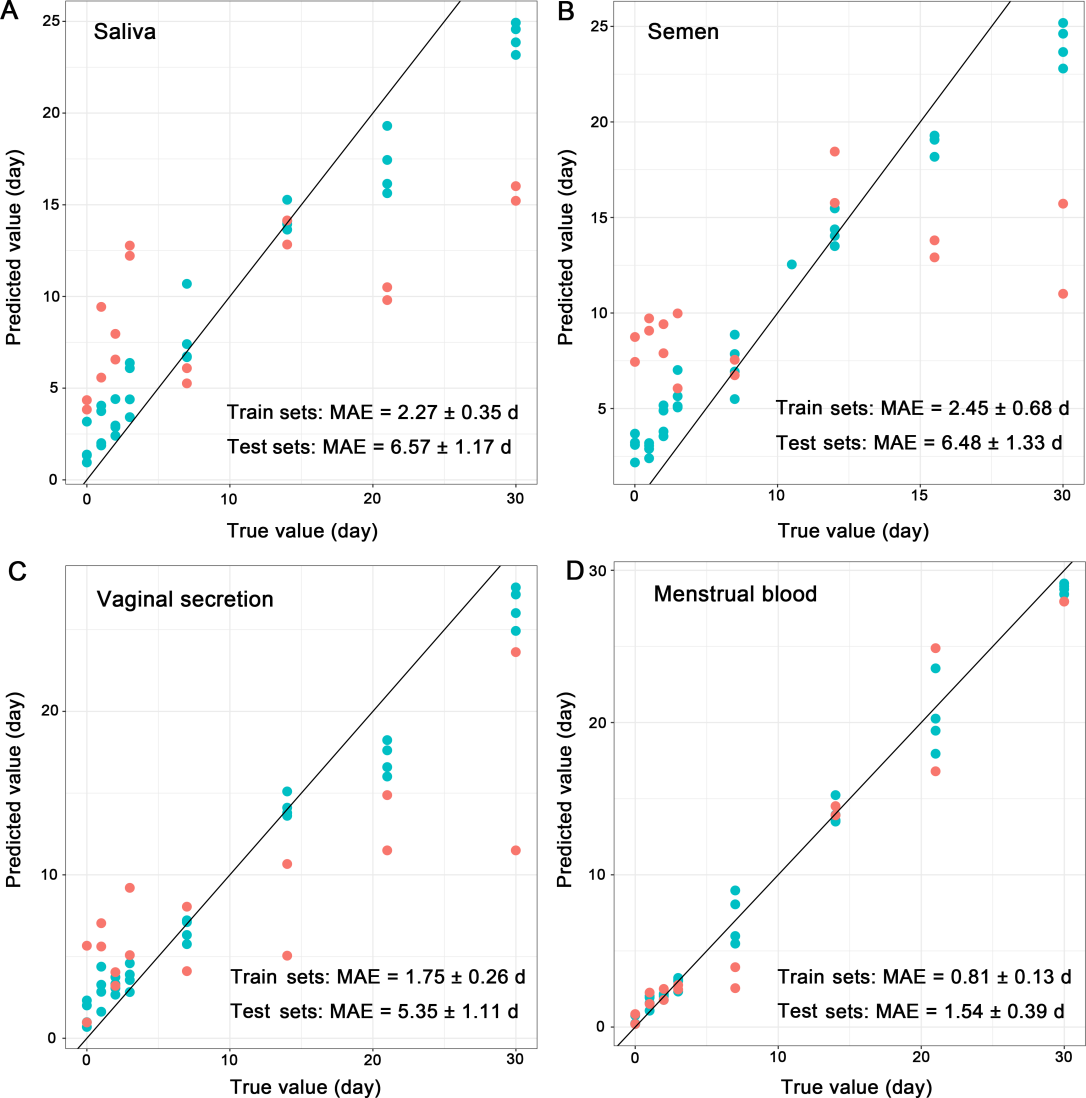
The predictive accuracies of TsD estimation performed with optimal biomarkers for saliva (**A**), semen (**B**), vaginal secretion (**C**), and menstrual blood (**D**) were shown using graph of predicted value versus true value. The line represents a perfect prediction of TsD. The cyan points represent the predictive results of train sets, and the pale red points represent the predictive results of test sets.

Then, the optimal biomarker sets were used to construct the final predictive model. The results showed that the accuracies of the final model in recognizing body fluids were the same as those of the initial model (training set: 100%, testing set: 98.43%; Fig. 5B). For the models for predicting TsD, the predictive results of the final models showed that the MAEs (mean ± SE) of menstrual blood (training set: 0.81 ± 0.13 d, testing set: 1.54 ± 0.39 d; Fig. 6D) were the lowest, followed by vaginal secretion (training set: 1.75 ± 0.26 d, testing set: 5.35 ± 1.11 d; Fig. 6C). The MAEs of saliva (training set: 2.27 ± 0.35 d, testing set: 6.57 ± 1.17 d; Fig. 6A) and semen (training set: 2.45 ± 0.68 d, testing set: 6.48 ± 1.33 d; Fig. 6B) were relatively higher, especially for the predictive results of the test sets.

## DISCUSSION

Microbiome-based stain analyses have attracted the attention of the forensics community as a promising tool for body fluid identification and TsD prediction (1). These two questions were investigated separately in previous studies. However, these two questions are not isolated but affect each other. Our current study provides the first assessment of the mutual influence between body fluid identification and TsD prediction and determines an optimal process for microbiome-based analyses that allows for identifying body fluids and estimating the TsD of stains simultaneously.

The compositions of the microbial community varied obviously among samples from different types of body fluids. The type of body fluids had a greater effect on the compositions of microbial communities in body fluid stains compared with TsD during the exposure experiment of 30 days. Furthermore, significant differences could still be observed after exposure for 30 days, although there were shifts in the microbial community for each body fluid.

Most of the test body fluid samples presented a taxonomic composition dominated by the Firmicutes phylum, in agreement with most previous studies (1, 21, 23–25). Only the microbial taxonomic profiles of semen were inconsistent with the research results (dominated by Proteobacteria) of Salzmann et al. (22). However, most studies on seminal microbiotas support our results (23, 26, 27). Of course, this does not mean that the result of Salzmann et al. (22) is incorrect. Human semen microbiome diversity shows spatial heterogeneity (28). Study populations from different regions may explain the difference in seminal microbial taxonomic compositions.

At the genus level, *Streptococcus* was the dominant taxon in the fresh salivary microbiome, followed by *Neisseria*, *Haemophilus*, *Rothia*, and *Prevotella*. In previous studies, these genera were considered common taxa in the saliva of healthy adults (29–31). These genera maintained their predominance throughout the duration of indoor exposure. *Streptococcus*, *Actinomyces*, and *Prevotella* increased and *Haemophilus* and *Neisseria* decreased along with TsD in relative abundance. These shifts were significant but slight. There was a lack of an obvious dominant genus in seminal microbiotas. Great variations in microbial compositions between individuals were observed. This result is in agreement with a previous study (23). It was difficult to observe a consistent shift at the genus level across the exposure experiment.

*Lactobacillus* was absolutely dominant at the genus level in vaginal secretion. The predominance was maintained across 30 days of exposure. Large amounts of *Lactobacillus* breeding in the vagina may produce an acidic environment to maintain vaginal health (32). Therefore, *Lactobacillus* was one of the dominant genera in fresh menstrual blood. Menstrual blood is a mixture of vaginal secretion and blood (25). The NMDS results showed that menstrual blood was most similar to vaginal secretion. However, there are still obvious variations between these two body fluids to distinguish them. The study of Song et al. (33) suggested that the relative abundance of *Lactobacillus* in the vagina decreased during menses (33). This is consistent with our results. Our samples of menstrual blood were collected from days 2–5 of menstrual cycle. Though, the previous study showed that the vaginal microbiome of most women remained relatively stable throughout the menstrual cycle (34), we are still unable to determine whether menstrual blood could be distinguished from vaginal secretion at the beginning or end of menses. The question will be explored in our further studies. In addition, the most obvious and regular succession of the microbial community was observed in the menstrual blood.

The Bray–Curtis distance between vaginal secretion and menstrual blood increased after exposure for 30 days, and the trend was completely contrary to other pairwise comparisons. In addition to cervical mucus (the main component of vaginal secretion), menstrual blood contains more blood (35). Although vaginal secretion and menstrual blood are excreted through the vagina, quite different nutrient substances determine different directions of succession of microbiology communities. Apart from that, exposure for 30 days diminished the uniqueness of microbial characteristics for each body fluid. The effects of the unique habitat for microbial communities disappeared, and the same exposure conditions and environmental microbial community might be related to convergence on microbial communities of body fluids (36). The previous studies showed that the microbial communities in different body parts of cadavers became similar during the decay process (37). In fact, deposition of body fluids could also be considered a decay process.

However, Bray–Curtis distances of pairwise comparisons at Day 30 still reached a high value to distinguish different body fluids. Dobay et al. (21) exposed body fluids (skin, saliva, peripheral blood, menstrual blood, vaginal fluid, and semen) to indoor conditions for 30 days, while the same samples were processed directly after extraction for comparison. They found that both types of samples were grouped by body site. PCA was performed separately for fresh and aged samples (21). Whether aged samples can be grouped with fresh samples from the same body site is unclear. The question is important. In actual cases, it is difficult to know exactly when the body fluid was deposited. Most studies use the microbial community of fresh body fluids to train a predictive model (2, 19, 20). In our study, the accuracies decreased at Day 21 and Day 30 when only fresh body fluids were used as training data. López et al. (20) trained a deep learning network with data from the HMP and found that the predictive accuracy decreased in aged mock casework samples compared with fresh samples. This suggested that the TsD of body fluids had an effect on the accuracy of body fluid identification based on microbial characteristics.

Therefore, the predictive model for body fluid identification constructed only with fresh samples may not be an optimal solution. Of course, this does not mean that an exact TsD must be known in advance before body fluid identification. The Bray–Curtis distances between samples from different types of body fluids were still significantly greater than those from the same type of body fluids without discriminating TsD. The model was constructed with the integrated data containing fresh and aged samples and reached a high identification accuracy. In other words, we can still recognize body fluids exactly even if we do not know the TsD. In turn, the Bray–Curtis distances between samples from different TsD values are almost completely coincident with the differences between samples from the same TsD without discriminating sample types. However, the differences were significant in each comparison of a specific body fluid. The accuracies of TsD prediction significantly decreased without discriminating the types of body fluids. This suggests that body fluids should be identified before estimating the TsD in microbiome-based stain analyses for forensics.

In addition, the predictive TsD of menstrual blood was more accurate than that of other body fluids. The predictive accuracies for saliva, semen, and vaginal secretion need to be further improved. Asaghiar et al. (38) evaluated two hypoxia-sensitive RNA markers based quantitative PCR (qPCR) to develop a predictive model of TsD for blood, saliva, and semen, resulting in an MAE of 4.2, 2.1, and 5 days of within 28 days of degrading at room temperature, respectively (38). Wang et al. (37) regressed a random forest model with salivary microbiota to predict TsD, resulting in an MAE of 1.41 days within 20 days of exposure (1). The models of these two studies were tested with training data. The predictive accuracies of our study were closed to or even better than those of the previous studies if we used the training sets for testing. However, the accuracies decreased significantly when the testing sets were tested. In general, the data used for validation should be distinguished from the training sets to avoid overfitting (39). A sustainable effect of individual difference was observed in the microbial communities of these three types of body fluids throughout the entire process of exposure. The variations in microbial communities caused by individual differences were even higher than those caused by TsD. A stable individual variation in the microbial community is adverse to TsD prediction but beneficial to individual identification.

The current study still lacked mock casework samples to test our conclusion. The complex environment of the crime scene may cause a deviation from our result. In addition, a longer study may produce a varying result. The predictive accuracies of TsD for saliva, semen, and vaginal secretion need further improvement by including a larger sample size or other biomarkers, such as mRNA. In addition, mixed bodily fluids with one or more body fluids are commonly encountered in crime scenes. However, mixed body fluids were not involved in the current study. Microbial source-tracking methods such as SourceTracker (40) and FEAST (41) can be applied to forensic science and provide a potential solution to identified mixed body fluids. These questions will be explored in our further study. However, the current study provided the first evidence to understand the mutual influence between body fluid identification and TsD prediction in microbiome-based stain analyses. Our study supplied a potential solution to recognize body fluid and estimate the TsD of stains simultaneously based on microbial characteristics for forensics and determined a relatively optimal process for codetection.

## MATERIALS AND METHODS

### Sample collection

Body fluid samples (including saliva, semen, vaginal secretion, and menstrual blood) were collected on sterile cotton swabs from health volunteers. All volunteers were recruited from Shanxi Medical University in March and provided written informed consent. Exclusion factors included taking antibiotics within 1 month. The study was ethically approved by the Medicine Institutional Review Board of Shanxi Medical University (No. 2021GLL049) according to the guidelines of the World Medical Association and the Declaration of Helsinki. Saliva and semen samples were first self-collected into sterile tubes and then transferred to sterile cotton swabs. Vaginal secretion and menstrual blood samples were self-collected from the vagina directly on sterile cotton swabs.

Then, all samples were placed on a lab bench and exposed to indoor conditions (temperature, 18.4–19.4°C; humidity, 30%–40%). Eight sampling time points (fresh, Day 1, Day 2, Day 3, Day 7, Day 14, Day 21, and Day 30) were set in our experiment. Each body fluid at each time point included six biological replicates corresponding to six volunteers. Saliva samples were collected from three males and three females. In total, 192 samples of body fluid stains were collected. All samples were collected and stored at −80°C before further processing.

### DNA extraction, library preparation, and sequencing

Total genomic DNA was extracted from body fluid stains using the DNeasy PowerSoil Kit (Qiagen, Hilden, Germany) following the manufacturer’s instructions. Universal primers (505F- GTGCCAGCMGCCGCGGTAA and 806R- GGACTACHVGGGTWTCTAAT) to the variable region 4 of the 16S rRNA gene, which contained Illumina adapters and barcode sequences for sequencing, were amplified. The amplification products were quantified using QuantStudio5.0 (Applied Biosystems, Foster City, USA) and pooled in equimolar concentrations. The pooled sequencing libraries were sequenced using the Illumina NovaSeq platform. Negative controls consisting of six sterile swabs were included during DNA extraction and sequencing.

### Data analysis

We obtained a cluster density of 1,130 k/mm^2^ and a passing filter of 88.9%. Totally, 16,335,500 sequences were generated. More than 97.07% of the bases have a Phred score above Q30. Raw data were quality filtered using fastp software (42). Then, the clean data were merged and dereplicated using VSEARCH (43) and USEARCH (44). The UCHIME algorithm (45) was applied to remove chimeric sequences. After trimming and filtering, 14,990,142 reads remained. USEARCH-UNOISE3 (46) was used to identify ASVs. The taxonomic annotation of the ASV sequence was obtained by alignment with the Silva (v138) database using BLAST (47).

The statistics in our study were analyzed using the R software platform (v3.6.0; http://www.r-project.org/). The taxonomic compositions of the samples were shown at the phylum and genus levels using the “ggplot2” package (48). Wilcoxon test was performed for the comparison between bacterial compositions of two groups, and the Kruskal–Wallis test was performed for the comparison between multiple groups. NMDS using the “vegan” package (49) was performed and visualized to assess the effects of body fluid origins and TsD on microbial community structures of body fluid stains. Permutational multivariate analysis of variance (PERMANOVA) based on Bray–Curtis distance was used to verify the result of NMDS in statistics. Within-group variation and between-group variation were calculated and compared based on the Bray–Curtis distance to test the discrimination of different grouping manners for samples. The Wilcoxon test with *P* values adjusted according to the Benjamini–Hochberg method (50) was performed to test the significance of differences in beta diversities among various groups. The correlation of microbial community similarities (a value of 1 minus Bray–Curtis distance was ln-transformed) with intervals between pairwise time points was tested by fitting a linear regression.

To measure the effect of the TsD of stain on body fluid identification, classification models for body fluid identification were constructed using the data that included all time points and the data of each time point. Then, the accuracies of the two classification models were compared. Similarly, regression models for TsD prediction were constructed using the data that included all types of body fluids and the data of each body fluid. The accuracies of the regression models were measured by using the MAE. The stratified random sampling method was employed to divide the data into training and testing sets at a proportion of 2:1 using the “sampling” package (51). The initial models were constructed with a training set using the random forest algorithm from the “randomForest” package (52). The value of “ntree” was set to 1,000. Bacterial ASVs were ranked in order of their feature importance. Furthermore, the random forest algorithm was used to select the optimal biomarker sets according to the order of importance based on the minimum error of 10-fold cross-validation by 100 iterations with five repeats. Each subset of training sets was partitioned based on individual for cross-validation. The final prediction models were constructed using the optimal biomarker sets.

## Data Availability

All sequence reads generated in this study have been deposited to the NCBI Sequence Read Archive (SRA) under BioProject accession PRJNA979326. Other data and code in this study are available from the corresponding author upon reasonable request.

## SUPPLEMENTAL MATERIAL

Supplemental figures spectrum.02480-23-s0001.pdf Fig. S1 to S5.

Supplemental material spectrum.02480-23-s0002.xlsx STORMS checklist.

ASM does not own the copyrights to Supplemental Material that may be linked to, or accessed through, an article. The authors have granted ASM a non-exclusive, world-wide license to publish the Supplemental Material files. Please contact the corresponding author directly for reuse.
