# Supplementary material for: Body fluids should be identified before estimating the time since deposition (TsD) in microbiome-based stain analyses for forensics
Source: Microbiol Spectr. 2024 Mar 12;12(4):e02480-23. doi: 10.1128/spectrum.02480-23 (PMC10986545; doi:10.1128/spectrum.02480-23)
Supplement: Supplemental figures — Fig. S1 to S5. [file spectrum.02480-23-s0001.pdf]

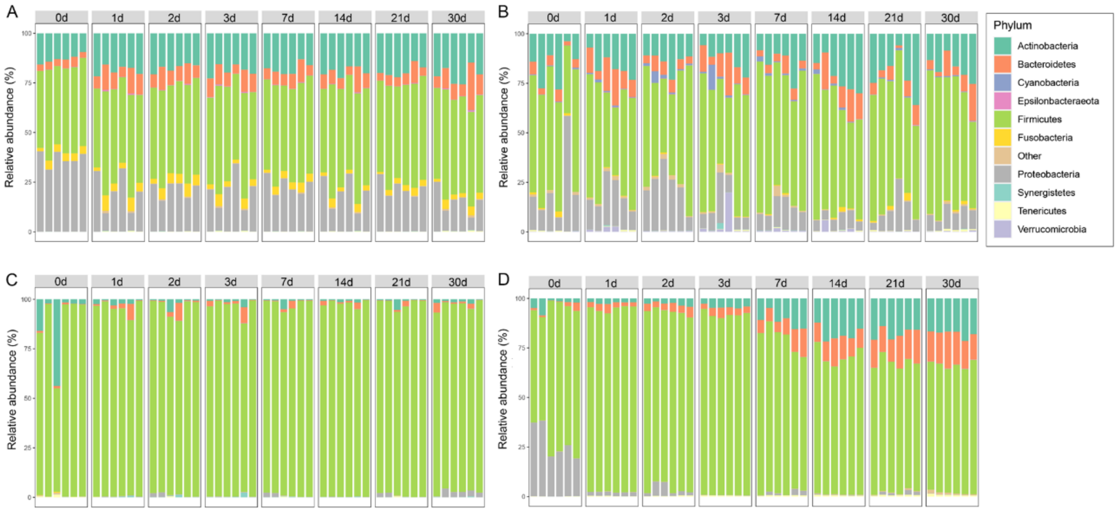

Fig. S1 Relative abundance of bacterial phylum in saliva (A), semen (B), vaginal secretion (C) and menstrual blood (D) across the exposure of 30 days.

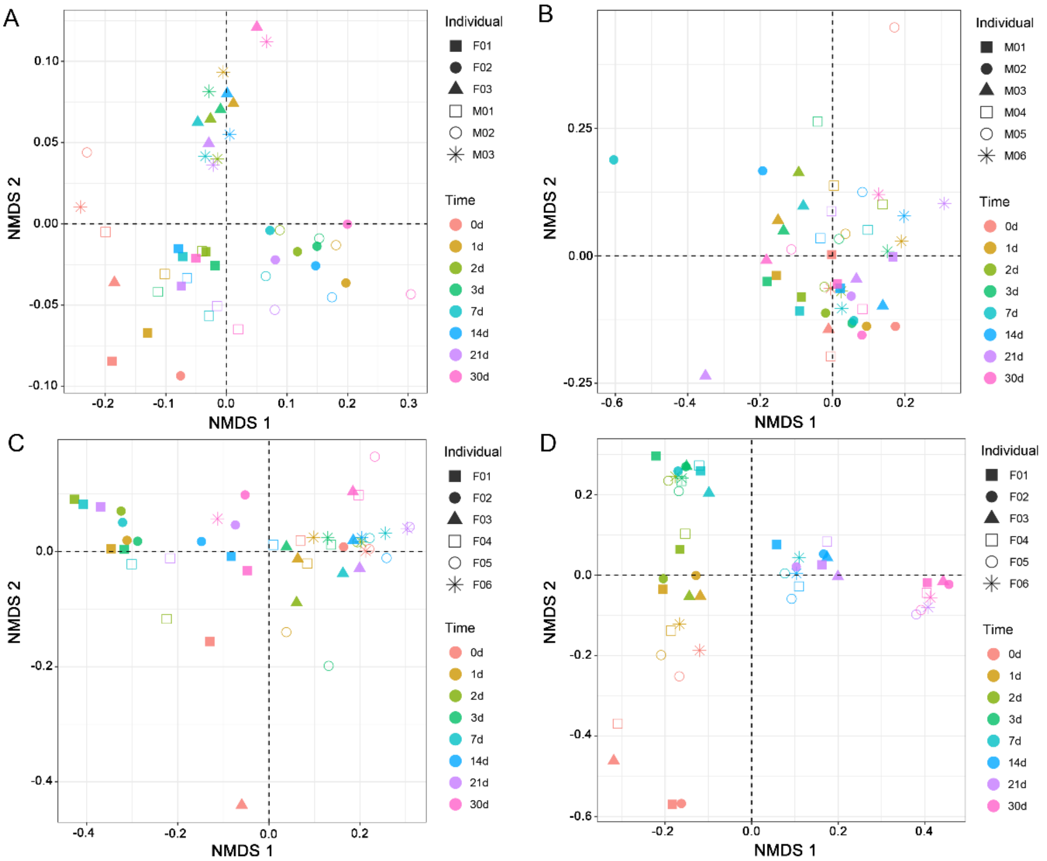

Fig. S2 Non-metric multidimensional scaling (NMDS) of bray-curtis distances between samples for saliva (A), semen (B), vaginal secretion (C) and menstrual blood (D).

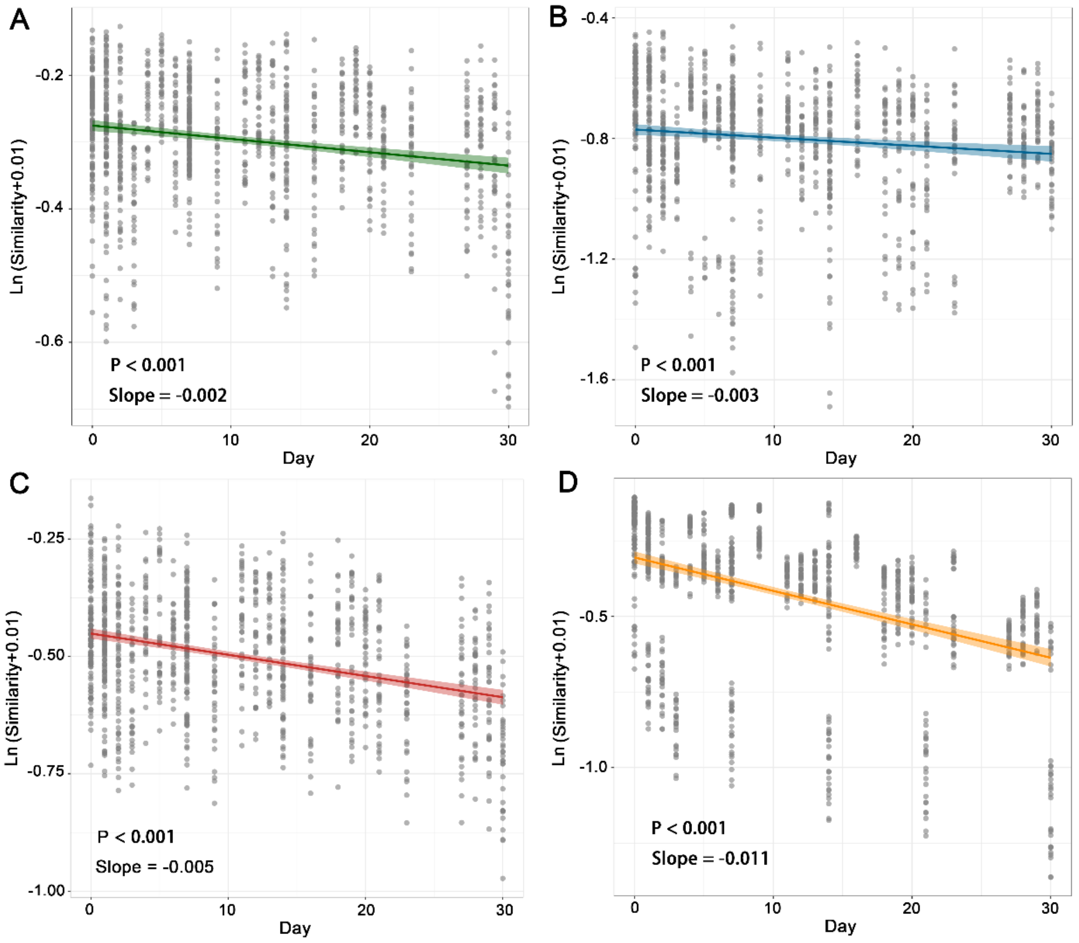

Fig. S3 Significant linear relationships between similarities (Ln transformed) of microbial communities against intervals of time since deposition were observed in saliva (A), semen (B), vaginal secretion (C) and menstrual blood (D).

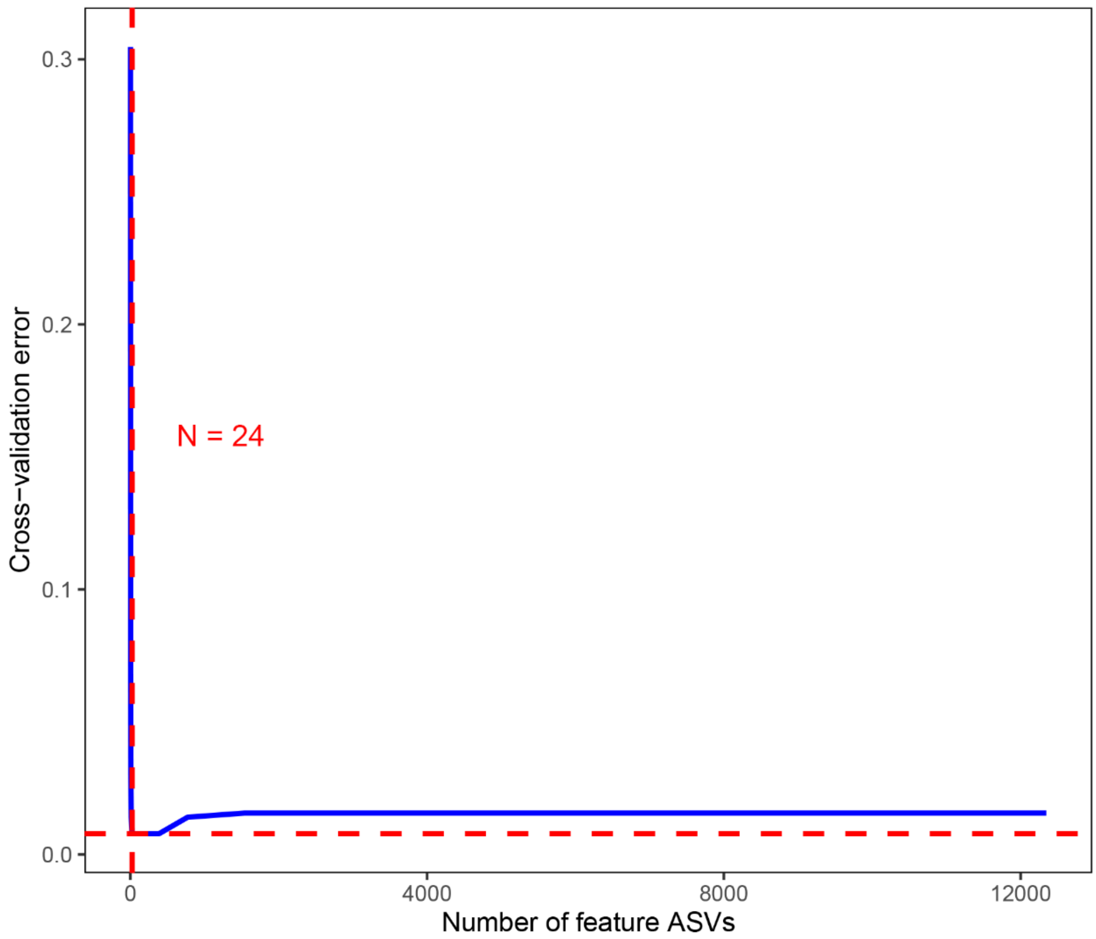

Fig. S4 The random forest algorithm was used to select the optimal biomarker sets to identify body fluids according to the order of importance based on the minimum error of 10-fold cross-validation by 100 iterations with five repeats.

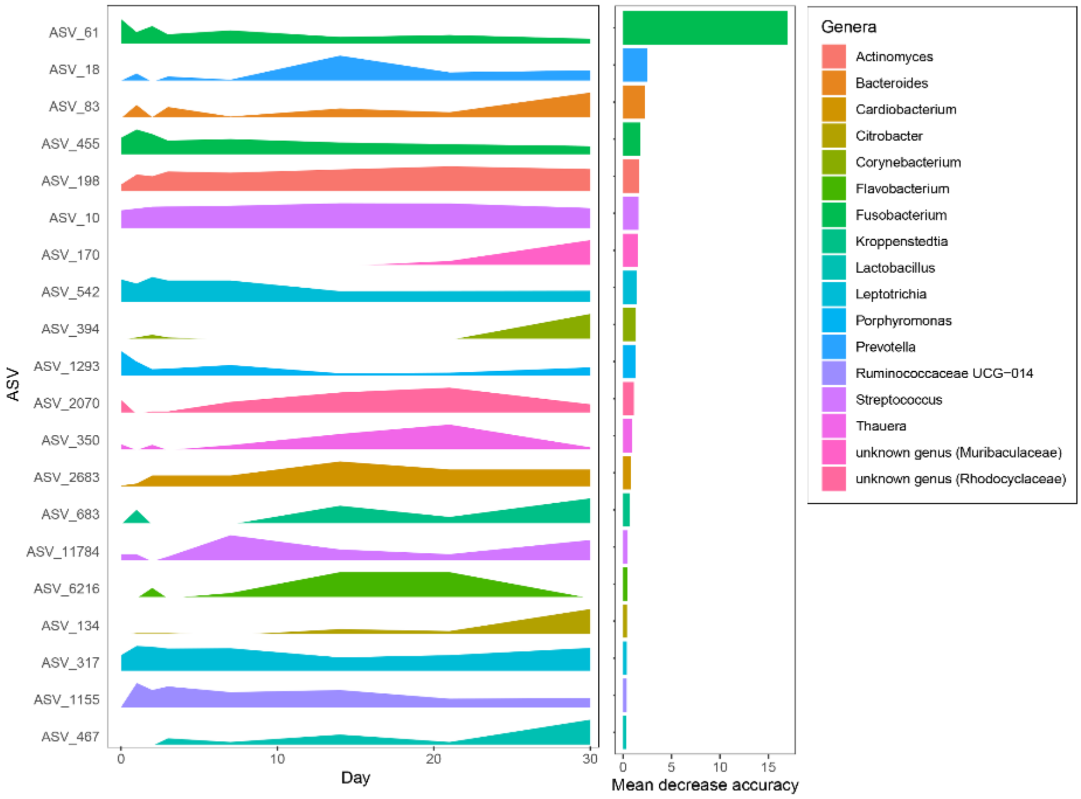

Fig. S5A The top 20 biomarker bacterial ASVs were identified by applying Random Forests regression of their relative abundances in saliva against TsD.

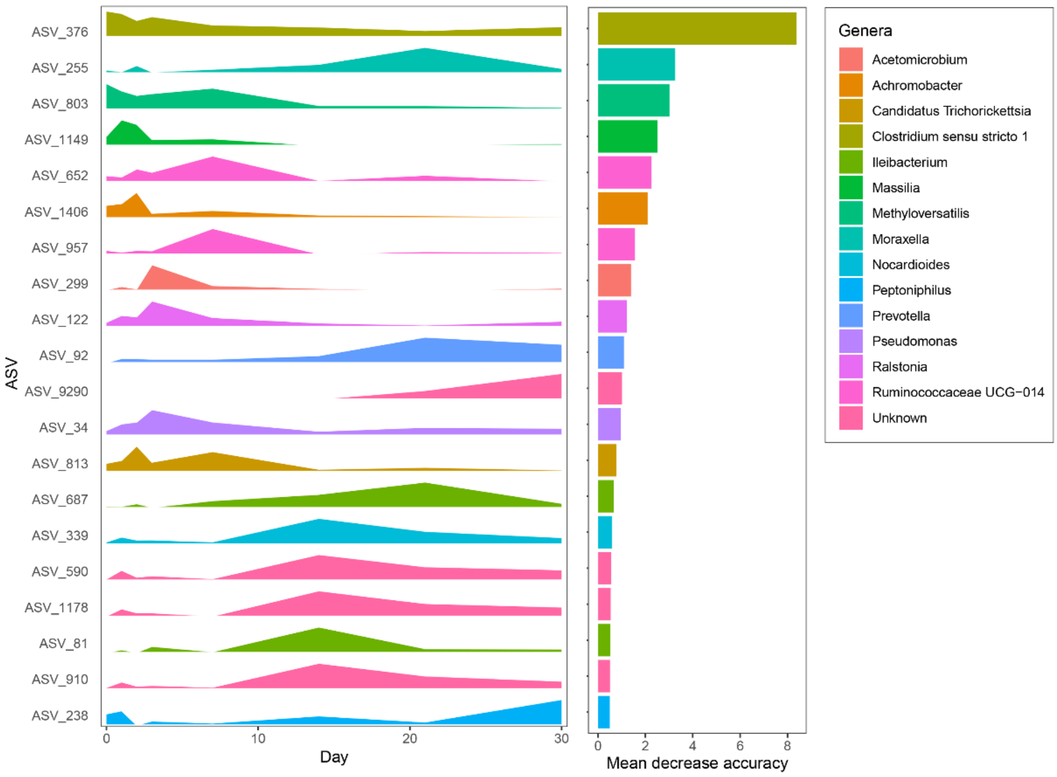

Fig. S5B The top 20 biomarker bacterial OTUs were identified by applying Random Forests regression of their relative abundances in semen against TsD.

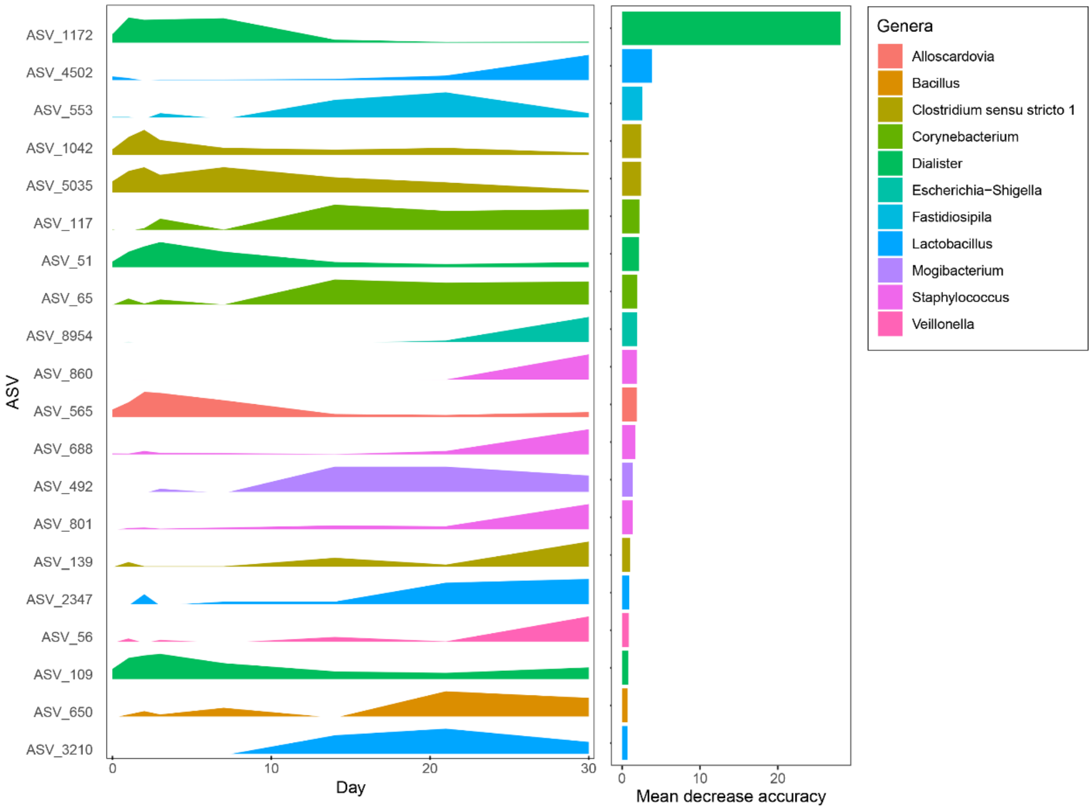

Fig. S5C The top 20 biomarker bacterial OTUs were identified by applying Random Forests regression of their relative abundances in vaginal secretion against TsD.

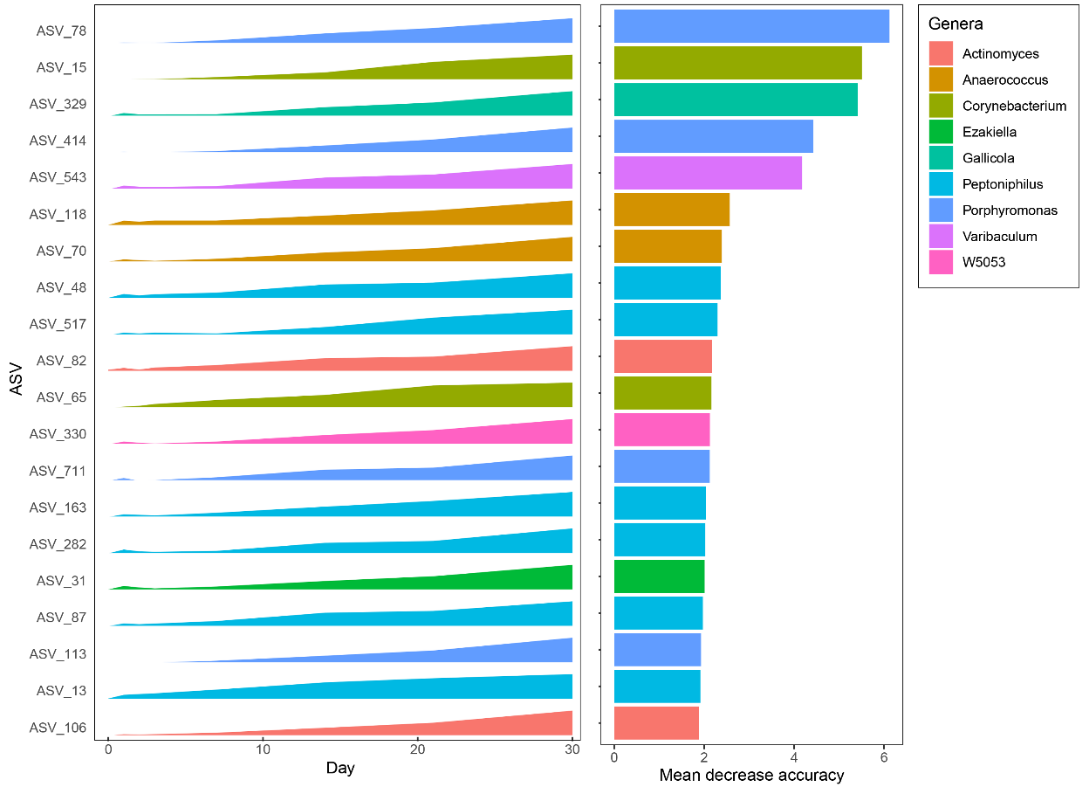

Fig. S5D The top 20 biomarker bacterial OTUs were identified by applying Random Forests regression of their relative abundances in menstrual blood against TsD.
